# Functioning and disability in recent research from Cameroon: a narrative synthesis

**DOI:** 10.11604/pamj.2017.27.73.12167

**Published:** 2017-06-01

**Authors:** Minal Ray, Lorena Wallace, Lawrence Mbuagbaw, Lynn Cockburn

**Affiliations:** 1 University of Toronto, Department of Occupational Science & Occupational Therapy, Toronto, Canada; 2 McMaster University, Department of Health Research Methods Evidence and Impact, Hamilton, ON, Canada; 3 Centre for the Development of Best Practices in Health, Yaoundé, Cameroon

**Keywords:** Disability research, community health, Cameroon, narrative synthesis

## Abstract

**Introduction:**

People living with disabilities in Cameroon face many barriers to daily functioning and social participation. However, there is limited research on disabilities and their impact. We sought to examine the research related to disability from Cameroon.

**Methods:**

We conducted a systematic review, bibliometric analysis, and narrative synthesis of research related to disability, functioning, and social participation from Cameroon published during 2005-2014. The articles were screened in duplicate to identify articles addressing impacts of disability on functioning. Disability was contextualized using the International Classification of Functioning, Disability and Health (ICF) framework. Data were analyzed narratively per identified themes using an inductive data-driven approach.

**Results:**

A total of 46 studies were included following full-text review of which 36 addressed non-communicable diseases and conditions, 7 addressed infectious diseases and 3 addressed neglected tropical diseases. Among ICF Activity and Participation Restrictions, work and employment was the highest reported category (19 studies), followed by intimate relationships (14 studies), and looking after one's health (8 studies). Among ICF Environmental Factors, societal attitudes were the highest reported category (21 studies), followed by health services, systems and policies (14 studies) and support and relationships (11 studies). Among other common themes, knowledge and awareness was the highest reported category (22 studies), closely followed by traditional beliefs (20 studies) and financial barriers (9 studies).

**Conclusion:**

There is a small body of primary research from Cameroon on disability. The main themes related to disability are stigma, limited knowledge and awareness, poor quality of care and hindered employment opportunities. Further efforts are required to investigate the complexities of living with a disability in Cameroon and strategies to enhance adequate participation in activities of daily life.

## Introduction

Globally, over a billion people live with disabilities [1]. Years of 'healthy life lost due to disability' is 80% higher in Africa than in high-income countries [2]. Cameroon is an African country with a population of over 22 million people [3]. It is bordered by six other countries: Nigeria, Chad, the Central African Republic, Equatorial Guinea, Gabon, and the Republic of Congo. Political and social strife in surrounding nations in recent years has put a strain on Cameroon. As of December 2014, over 200,000 people had fled their home nations crossing Cameroonian borders to find refuge [4]. Cameroon has a rich cultural tradition but a poor economy. The difficulties Cameroon faces include high unemployment rates, political challenges, insufficient infrastructure, a resource-lacking educational system, and a poor healthcare system which is struggling to meet the needs of its growing population [5, 6]. Cameroon ranks 152 on the United Nations (UN) Human Development index [7], indicating that the rate of desirable life outcomes of the people of Cameroon is below the world average. In 2010, there were 1.1 physicians per 10,000 people [8]. Although life expectancy at birth is rising, it is currently only 55.7 years [7] compared to the average global life expectancy of 71 years [9]. Such factors negatively affect the health and well-being of the people of Cameroon.

The current body of research on disability in low-income countries such as Cameroon is quite small [10]. Most of the studies that do exist are concerned with prevalence and patterns of disability and do not address the impact of disability. Disability related research priorities for the Cameroonian health and social services systems have not been clearly stipulated, no funds are allocated to disability research, and little attention is paid to disability and rehabilitation programming. The lack of research and relevant reviews make it difficult for clinicians, educators, and policy makers to locate and evaluate empirically based evidence relevant to the population. The provision of a comprehensive review of disability-related research in Cameroon could guide practice, policy, and future research to improve the lives of the general population and people living with disability (PLWD). A combination of systematic review and bibliometric review methods alongside a narrative synthesis were chosen to address this need. The International Classification of Functioning, Disability and Health (ICF) proposes that disability can be understood as the result of complex interactions between health conditions and personal, environmental, and occupational factors impacting social participation and day-to-day functioning [11]. The ICF provides an integrated biopsychosocial framework for understanding human functioning and the factors related to disability [11]. According to the ICF, disability involves dysfunction between three levels: impairments, activity limitations, and participation restrictions [11]. The ICF has been adopted and used around the world in many different contexts, including disability research.

The ICF is becoming more widespread as researchers strive towards common understandings and language for discussions of health and disability, and therefore the use of the ICF framework is favorable for research on disability and functioning in Cameroon. For this study, we used the ICF as an organizational framework to understand the impacts of disability on functioning and the contextual factors that impact the relationship between disability and functioning. Because the focus of this paper is on Activity and Participation Restrictions that result from disability and Environmental Factors that impact disability and functioning, we do not report on or discuss the Impairments in [body] Structure and Function, which are also part of the ICF framework [11]. The purpose of this review is to inform and guide research priorities, policy development, funding allocations, and evidence based-practice in Cameroon. The specific objectives were: (1) To access, categorize, and synthesize the disability research focused on social participation and functioning from Cameroon published during 2005-2014; (2) To examine and synthesize the impact of disability on the functioning of Cameroonians, including activities of daily living and social participation; (3) To identify and describe the environmental factors which are most commonly studied, such as stigma and barriers to accessibility.

## Methods

**Data collection and search strategy:** A comprehensive search of major and relevant databases available through the University of Toronto library (https://onesearch.library.utoronto.ca/) was conducted in January of 2015. Literature was obtained from the following databases/providers: Biomed Central, Elton B. Stephens Co. (EBSCO), Francis, Journal Storage, Popline, Project Muse, Proquest, PubMed, Social Science Abstracts, Scopus, Web of Science, and OVID databases. The tables of contents of two journals published in Cameroon, the African Journal of Integrated Health and African Health Sciences, were also hand searched.

**Data management and data sharing:** Citations were downloaded into the reference management system Zotero (https://www.zotero.org/). A pre-developed extraction table in Microsoft Excel was used to record relevant information when conducting in-depth analyses of articles that fit the inclusion criteria.

**Screening:** Title and abstract were reviewed to ensure that articles met the following inclusion criteria: Indexed between January 1, 2005 and December 31, 2014; primary health-related research focusing on humans living in Cameroon; published in English or French; Mixed-method, observational, qualitative, and quantitative studies; articles with analysis of primary or secondary data collected in Cameroon. Studies using secondary data were included if an original analysis was conducted on this data (e.g. articles that conduct analysis on data collected from Demographic and Health Surveys studies were acceptable for inclusion); in multi-country studies, where Cameroon was one of two or more countries, the study was included if information and results regarding Cameroon could be extracted independently from data about other countries. We excluded: Publications such as commentaries and letters to the editor that did not involve direct contact with participants; animal, plant and basic pharmaceutical/lab studies; secondary studies (i.e. reviews); masters and Doctoral theses. From the list of health-related research from Cameroon, we screened for studies that included information on disabilities. First, the Zotero library (containing the list of health related research from Cameroon for a ten year period), was thoroughly screened in duplicate by MR and LW via title and abstract reviews to identify all studies that potentially addressed disability and functioning. Only studies that had a focus on a health condition were included. A 'health condition' was defined as a disease or disorder affecting a person's mental or physical health or daily functioning. Such conditions include: Non-communicable diseases and conditions (including mental health disorders), infectious diseases, and neglected tropical diseases. These groupings were identified based on those found within Cameroon's Sectoral Strategy for Health document [12]. Identified studies were then carefully read in full text to further identify studies that addressed impacts of disability (i.e. ability to function, to live one's life, or carry out daily occupation). A third author (LM or LC) was invited to arbitrate when a consensus could not be reached.

**Data analyses:** Using an inductive, data-driven approach, the results sections of included articles were systematically searched for data relevant to the ICF categories related to activity and participation. Additional potential themes were drawn from data. Raw data was extracted directly from articles, and grouped using the ICF categories and addition working themes not captured by the ICF categories. The themes were verified by the team. Descriptive statistics were conducted using counts and percentages. Raw data fitting each category or theme was narratively analyzed and information was synthesized. Content for each identified theme is reported narratively.

## Results

Our search retrieved 8624 studies of which 3009 were remaining after deduplication. Eighty-five were eligible for full text review, of which 46 were included in our narrative synthesis.

**Figure 1:**
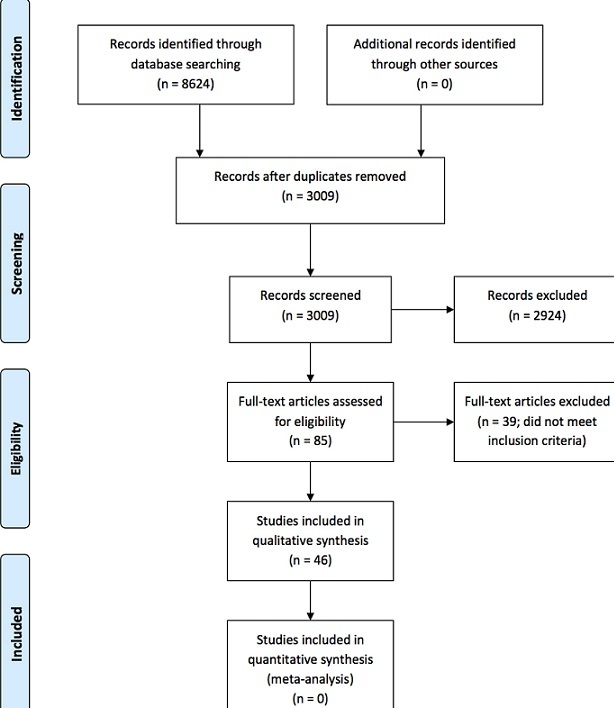
Flow diagram of screening and selection

**Figure 1** is a Preferred Reporting Items for Systematic Reviews and Meta-Analysis (PRISMA) flow diagram detailing our screening process [13].

**Characteristics of included studies:** A total of 46 studies were included following full-text review of which 36 addressed non-communicable diseases and conditions, 7 addressed infectious diseases and 3 addressed neglected tropical diseases. There was a predominance of quantitative survey approaches and few phenomenological or anthropological studies. Table 1 provides a brief description of the included studies and outlines the location, design, and disease or condition covered. The studies came from many parts of Cameroon, with more from the Central, North West, and South West regions. There was a dominance of studies addressing neurological conditions due to the relatively large number of articles addressing epilepsy.

**Table 1:** Characteristics of studies included in this review including the location, design and disease or condition covered

Study ID	Region	Study Design	Disease/Condition
Allotey 2007	Centre	Mixed methods	Epilepsy
Bain 2013	North West	Cross-sectional	Epilepsy
Boyer 2011	Across Cameroon	Retrospective	HIV and adherence to medication
Boyer 2012	Littoral	Cross-sectional	HIV
Bremer 2010	North West	Qualitative	Physical disabilities and reproductive health
Cubo 2014	Littoral	Cross-sectional design	Parkinson’s Disease, anxiety, depression
Ekotarl 2007	Centre	Cross sectional	Cancer
Fernande 2014	Across Cameroon	Qualitative	Physical disabilities
Gaynes 2012	North West	Cross-sectional	HIV and depression
Grietens 2012	Centre	Mixed methods	Buruli Ulcer Disease
Heinserling 2005	South West	Qualitative study	N/A
Hofer 2014	North West	Cross-sectional	Generative concerns
Jacobi 2013	South West	Cross-sectional	HIV
Jacobi 2013	South West	Cross-sectional	HIV
John 2009	South West	Cross-sectional	Poverty
Keugoung 2013	Far North	Mixed methods	Suicide
Kiani 2009	North West	Cross sectional	Women with disabilities
Labhardt 2010	Centre	Cross-sectional	Care seeking attitudes
Labhardt 2010b	Centre	Before and after study	Mental illness
L'akoa 2013	Centre	Cross-sectional	HIV, depression
Louis 2013	Littoral	Cross-sectional	Mental illness
Marcellin 2010	Across Cameroon	Cross-sectional	HIV
Melgar 2012	Across Cameroon	Retrospective	Depression
Menick 2005	Centre	Retrospective	Mental illness and pregnancy
Menick 2006	Centre	Retrospective	Mental illness and rehabilitation
Menick 2006	Centre	Retrospective	Depression
Menick 2009	Centre	Retrospective	Mental illness and homicide
Nanfosso 2010	Centre/Littoral	Cross-sectional	Fertility
Njamnshi 2009	Centre	Cross-sectional	Epilepsy
Njamnshi 2009a	North West	Cross-sectional	Epilepsy
Njamnshi 2009b	South West	Cross-sectional	Epilepsy
Njamnshi 2009c	South	Cross-sectional	Epilepsy
Njamnshi 2009d	North West	Cross-sectional	Epilepsy
Njamnshi 2009e	Centre	Cross-sectional	Epilepsy
Njamnshi 2010	North West	Cross-sectional	Epilepsy
Njamnshi 2010b	North West	Cross-sectional	Epilepsy
Njamnshi 2010c	South West	Cross-sectional	Epilepsy
Oye 2006	South West	Cross-sectional	Visual impairment
Oye 2007	South West	Cross-sectional	Visual impairment
Pence 2012	North West	Cross-sectional	HIV and depression
Pence 2014	North West	Prospective cohort	HIV and depression
Rosenburg 2012	Across Cameroon	Cross-sectional	HIV
Suzan-Monti 2011	Across Cameroon	Cross-sectional	HIV
Tebeu 2008	Far North	Cross-sectional	Cervical cancer
Touko 2010	Centre	Cross-sectional	Hearing impairment and HIV
Zamo-Akono 2013	Across Cameroon	Cross-sectional	Disability

**Figure 2:**
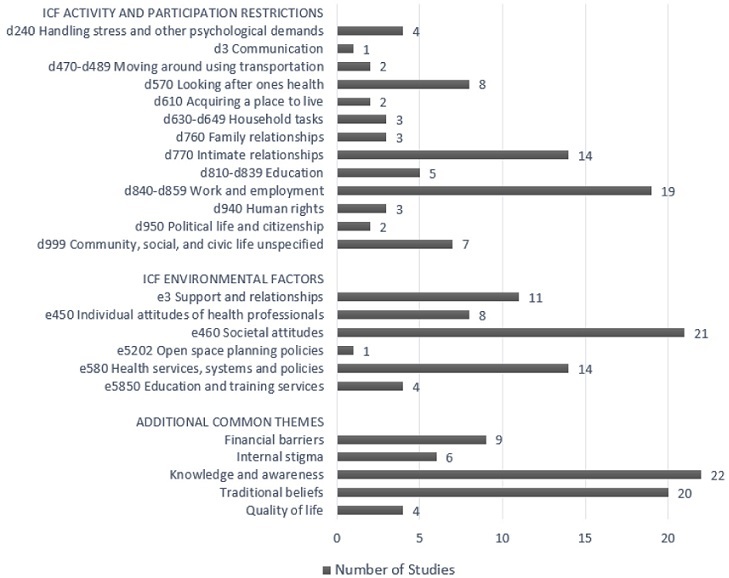
Themes addressed in functioning and disability research from Cameroon

**ICF classification:** in addition to the ICF categories, we identified other themes that became apparent during this analysis. addresses the disease groupings in more detail by looking at the disease conditions that fall within each grouping. The ICF Activity and Participation Restrictions, ICF Environmental Factors, and additional common themes that were mentioned at least once are plotted in Table 2 (not weighted). Figure 2 quantifies the number of studies that addressed each of the ICF categories and additional common themes. Among ICF Activity and Participation Restrictions, work and employment was the highest reported category (19 studies), followed by intimate relationships (14 studies), and looking after one's health (8 studies). Among ICF Environmental Factors, societal attitudes was the highest reported category (21 studies), followed by health services, systems and policies (14 studies) and support and relationships (11 studies). Among other common themes, knowledge and awareness was the highest reported category (22 studies), closely followed by traditional beliefs (20 studies) and financial barriers (9 studies).

**Table 2:** International classification of functioning, disability and health (ICF) categories identified in Cameroonian disability research; an “x” indicates that at least one study addressed the ICF category or theme: some studies addressed more than one condition

Disease/Health Condition			*Non-Communicable Diseases and Conditions*						*Infectious Diseases*	*Neglected Tropical Diseases*
			A	B	C	D	E	F		
d240	**ICF Activity and Participation Restrictions**	Handling stress	**x**						**x**	
d3		Communication			**x**					
d470-d489		Transportation		**x**		**x**				
d570		Self-health-care	**x**				**x**		**x**	**x**
d610		Acquiring a place to live	**x**						**x**	
d630-d649		Household tasks		**x**	**x**	**x**				
d760		Family relationships		**x**	**x**				**x**	
d770		Intimate relationships		**x**		**x**			**x**	
d810-d839		Education		**x**	**x**	**x**				
d840-d859		Work and employment	**x**	**x**	**x**	**x**			**x**	
d940		Human rights			**x**	**x**			**x**	
d950		Political life and citizenship			**x**	**x**				
d999		Community, social, and civic life	**x**	**x**	**x**	**x**	**x**		**x**	
e3	**ICF Environmental Factors**	Support and relationships	**x**	**x**	**x**	**x**		**x**	**x**	**x**
e450		Attitudes of health professionals		**x**		**x**	**x**		**x**	**x**
e460		Societal attitudes	**x**	**x**	**x**	**x**	**x**		**x**	
e5202		Open space planning policies				**x**				
e580		Health services, systems and policies	**x**			**x**	**x**	**x**	**x**	
e5850	
		Education and training services	**x**	**x**						
	**Additional Themes**	Financial barriers	**x**	**x**		**x**	**x**		**x**
		Internal stigma		**x**	**x**	**x**		**x**	**x**	
		Knowledge and awareness	**x**	**x**	**x**		**x**	**x**		**x**
		Traditional beliefs	**x**	**x**		**x**	**x**	**x**		**x**
		Quality of life		**x**	**x**					

A = Mental Health and Mental Illness B = Neurological Conditions C = Sensory Conditions D = Physical Disabilities E = Metabolic and Cardiovascular Conditions F = Cancers

### Non-Communicable Diseases and Conditions

*Mental health and mental illness:* Eight articles that directly addressed mental health/illness met our criteria. These articles discussed the impact of mental disability on functioning. Four articles focused on depression [14–17]. Three of these studies addressed depression in people living with HIV and AIDS (PLWHA). Topics included the prevalence, predictors, or correlates of depression, and the efficacy of intervention. Two articles included participants with psychosis/schizophrenia and other mental illnesses [18, 19]. One article examined suicide [20]. No studies were found that discussed indigenous conceptualizations of mental health or mental illness, anxiety disorders, personality disorders, or eating disorders. Knowledge and awareness of mental health in the public and in health systems was addressed in three studies. St. Louis & Roberts [21], looked at attitudes about mental illness in general and discussed stigma of mental illness: compared to Canadians and Americans, people in Cameroon had more negative impressions of mental illness, were less likely to endorse advocating for treatment, were more likely to engage in stigmatizing behaviour, and viewed people with mental illness as unable to function in daily living. Keugoung et al [20] and Gaynes et al [14] assessed the capacity of district health systems to deliver quality mental health care. Results suggest that most people with depression or other mental illness did not seek biomedical treatment, there was a lack of specific guidelines for treatment of mental health disorders among primary healthcare facilities, and a general lack of professionals trained in mental health care [14, 20]. Regions with millions of inhabitants (such as the Far North region) did not have doctors trained in psychiatry [20]. People with severe mental illness living in rural districts were referred to a psychiatric hospital in Yaoundé and follow-up communication between doctors in this hospital and the patient´s local doctors did not occur [20]. Knowledge of mental health/illness was also limited. Eighty-seven percent of consulting nurses interviewed by Keugoung et al [20] were not able to name any signs of depression and did not know it was associated with suicide. No other studies were found that addressed health worker capacity to address other disabilities.

*Neurological conditions:* The literature on neurological conditions was dominated by studies addressing knowledge, attitudes, and perceptions of epilepsy. Of a total of 12 studies in this category, 11 addressed epilepsy [22–32] and one addressed Parkinson's Disease (PD) [33]. Studies surveyed the public, secondary school students, and healthcare providers in different health districts across Cameroon to determine prevalence of stigmatizing attitudes towards people living with epilepsy (PLWE). Discrimination against PLWE was the highest when concerning the prospect of marriage. While stigma was present among medical professionals, stigma was greatest amongst traditional healers. In Badissa Village, lower rates of stigmatizing attitudes were attributed to an epilepsy education and treatment program established 10 years prior to the study [28]. In contrast, districts which lacked an epilepsy education and treatment program at the time the studies were conducted (Akwaya Health District, Batibo Health District, Fundong Health District, Kumbo West Health District, Ebolowa and Sangmelima) showed high rates of stigmatizing attitudes. All 10 studies found that predictors of negative attitudes towards PLWE were the beliefs that epilepsy is contagious and a form of insanity. In contrast to studies that focused on quantifying the prevalence of stigmatizing beliefs, Allotey & Reidpath [22] and Cubo et al [33] qualitatively focused on the impacts of epilepsy and PD, respectively, on functioning. PLWE reported a lack of supportive relationships and disruptions to meaningful family relationships [22]. Caregiver burden was a factor in caring for PLWE as well as people with PD [22,33]. In regards to intimate relationships, hypersexuality was found among people with PD [33]. Restrictions placed on sexual intercourse by medical doctors and healers affected self-esteem for males and contributed to internalized stigma [22]. Restrictions placed on PLWE by medical care providers and cultural beliefs resulted in the internalization of a 'sick' role, which posed barriers to participation in productive activities [22]. PLWE reported being subject to high levels of discrimination which hindered their participation in community, social and civic life, and reduced their “social value” [22]. PLWE were excluded from employment opportunities, resulting in difficulties supporting their family and affording medications for their condition; 40/42 participants reported compliance with medications only when finances were available [22]. Furthermore, 30/42 participants reported interruptions in education due to seizures; some were expelled from school because of them [22]. Participation in domestic life was also hindered. For example, women were advised to stay away from open fires which resulted in an inability to participate in cooking - an important role for women in Cameroon [22].

*Sensory conditions:* The only sensory condition that was studied with regard to the impact on functioning was the loss of hearing [34, 35]. Studies addressing vision loss reported only prevalence and did not discuss impacts on functioning [36, 37]. De Clerck [34] addressed the low quality of life of deaf individuals. She found that societal attitudes were highly discriminating and questioned the ability of deaf individuals to participate in activities associated with 'full personhood' in Africa such as employment, marriage, having children, and supporting a family. Deaf individuals reported internalized stigma, negatively affecting perceptions of themselves and other deaf people [34]. Access to employment opportunities was restricted due to a lack of education and low literacy rates among people who are deaf [34, 35]. Jobs that were obtained were often difficult and precarious [34, 35]. The lack of employment opportunities for deaf individuals resulted in financial barriers and further marginalization [34]. Deaf individuals lacked supportive relationships, were often left out of the family [34], and experienced human rights violations such as sexual abuse [34]. Many deaf individuals were not aware of the Cameroonian legal system and lacked the knowledge to self-advocate [34, 35].

*Physical disabilities:* Of five studies addressing the impacts of physical disabilities, Bremer et al [38], Fernande and Paul-Iva [39] and Kiani [40] addressed participation restrictions for people living with mobility impairments (PLWMI). Kiani [40], Nanfosso and Zamo-Akono [41] and Zamo-Akono [42] addressed employment restrictions. All five studies found that having a physical disability made it difficult to participate in employment [38–42]. For PLWMI, participation in employment was impacted by a lack of access to education due to the physical inaccessibility of schools and financial barriers [38–40]. PLWMI reported reduced availability of health services [38–39] as medical consultation and transportation were costly and taxis often refused to provide service [38].Participants reported that healthcare workers were not informed nor sensitive to the needs of PLWMI, negatively impacting quality of service [38]. PLWMI reported a lack of supportive relationships and stigmatizing societal attitudes [38, 40]. Participation was restricted in their community, social, and civic life due to the inaccessibility of public spaces and societal attitudes [39]. Beliefs, such as disabilities are caused by divine punishment, can lead PLWD to avoid being in public spaces [39]. Participation in domestic life was difficult for PLWMI due to activity limitations for tasks such as fetching water and cooking, and participation in social life, such as marriage [38]. Fernande and Paul-Ivan [39] discussed the violation of rights of that PLWD face. Even though laws regarding accessibility exist in Cameroon, they are rarely adhered to and PLWD are left out of decision-making processes regarding open space planning and policies [39].

*Metabolic and cardiovascular conditions:* Six articles regarding metabolic and cardiovascular conditions were included. Of these, four discussed knowledge, attitudes, or awareness of risk factors [43–46]. Four addressed the impact of these conditions on participation and functioning by examining management of disease through activity and lifestyle modifications [43–45, 47, 48]. These authors stressed the importance of reducing risk factors for diabetes and cardiovascular conditions. Kiawi et al [43] found that knowledge of these topic areas in Douala, Yaounde, Bamenda and Garoua was limited, and diabetes was considered to be 'the sugar disease,' with its cause attributed solely to eating sweet foodstuffs. Diabetes was commonly self-diagnosed, using methods such as tasting one's urine. If the urine tasted sweet, it was assumed to be because of diabetes [47]. Diabetes was not seen as a big threat to ones' health and not viewed as a chronic condition [43]. Awah et al [44] reported that a reduction in risk factors was difficult due to the lack of a national policy programme on non-communicable diseases combined with low awareness of the need for a reduction of risk factors. Perhaps some progress was made as the results of a study conducted by Fezeu et al [46] indicated that individuals residing in the Bamenda health district had adequate general knowledge about risk factors, symptoms, treatments, and complications of diabetes. Examining societal beliefs that may be correlated to diabetes, Kaiwi et. al. found that poverty is associated with thinness and with walking as a means of transportation. As a result, having a larger body size was valued and walking as a form of exercise was not [43]. Hence, obesity, along with physical inactivity and poor diet, was often not believed to be associated with diabetes. Traditional beliefs regarding the cause of diabetes, such as that the disease is a result of a social transgression of the patient or punishment from ancestors for neglecting culturally important rituals, may lead people to seek traditional healers [47]. In addition, many patients did not have the financial means to follow recommended diets [44], to access biomedical services and medicines [48], resulting in reliance on more affordable traditional options [43].

*Cancer:* the search revealed two studies that addressed cancer. Neither addressed the impacts of cancer on participation in daily life, however they identified key factors resulting in delay in seeking care [49] or an unwillingness to seek care [50]. Inadequate health services and lack of knowledge and awareness about cancer combined with the role of traditional beliefs were discussed in both studies.

**Infectious diseases:** Despite the large number of studies focusing on HIV infection, few studies examined disability related to HIV. Seven studies about infectious diseases addressed disability or functioning: six addressed HIV [51–56] and one focused on tuberculosis (TB) [57]. The main activity and participation restrictions faced by PLWHA identified in these six studies included: human rights violations, acquisition of necessities, engaging in work and employment; and engaging in intimate relationships. Jacobi et al. reported 12% of participants experienced violations of their human rights due to their HIV status [53]. Participants in this study reported being forced to change their residence or being unable to rent housing and some believed it was due to their HIV status [53]. Twenty-three percent of the participants lost their job at least once in the past 12 months and more than 40% of believed it was due to their HIV status [53]. Engaging in intimate relationships was hindered for PLWHA as 25% of participants isolated themselves and decided to refrain from marriage, sex, or having children due to internalized feelings of shame or guilt, self-blame, and low self-esteem [53]. Boyer found that PLWHA were discriminated against within their family [51]. Suzan-Monti et. al reported that among 1673 participants, 15% did not disclose their serostatus to their main partner, however, individuals who knew someone else with HIV in their family or social circle were more likely to disclose their HIV status [56]. PLWHA with stronger social supports reported better physical and mental health [52]. Access to healthcare and quality of care received was impacted by inability to pay for treatment, individual attitudes of healthcare professionals, and lack of services. In their study on HIV-related stigma, Rosenburg et al. reported that Cameroonian nursing students witnessed nurses providing poorer quality of care to a PLWHA than to other patients. This study reported that not only were PLWHA discriminated against by nurses, but nurses caring for PLWHA were also discriminated against [55].

**Neglected tropical diseases:** We identified only three studies on the impact of the impairments caused by neglected tropical diseases on functioning. Two addressed the impacts of Buruli Ulcer Disease (BUD) [58, 59] and one paper addressed onchocerciasis [60]. These studies focused mainly on knowledge and awareness of the diseases, as well as factors influencing treatment-seeking behaviours. Amuyunzu-Nyamongo et al found that children in Cameroon have greater knowledge and awareness of onchocerciasis and its treatment than children in Uganda and Nigeria [60]. The papers on BUD discussed knowledge of perceived etiology of BUD, factors influencing the form of treatment sought, and financial/social costs of treatment. Grietens et al found that study participants believed that even if BUD can be attributed to natural causes (i.e. 'microbes'), there were also beliefs that occurrence was influenced by witchcraft or patients' disregard of societal rules. These studies indicate that a large percentage of Cameroonians with BUD alternate between the use of traditional and biomedical treatment or utilize both types of treatment [59]. Cost of treatment and the unequal balance of power between doctor and patient were found to have a larger influence on treatment seeking behaviour than beliefs in a treatment type [59]. One study showed that the median cost burden of BUD amounted to 25% of patients' annual household earnings [58] influencing patients to choose traditional healing in an attempt to cut treatment costs [59]. Families of BUD patients tried to cope with costs by relying on friends and relatives to assist them financially. In 63% of households, coping strategies included the social isolation of the BUD patient [59].

## Discussion

**Methodology and process:** Use of the ICF was helpful because it provided the researchers with a list of categories that look at functioning from a wide lens, however there were still aspects that were missing, and additional themes were identified that were not included in the ICF categories. The broad categories of the ICF have been used effectively in other reviews of disability and rehabilitation [61]. One benefit of the use of the ICF framework is that its standardized categories allow for these findings to be compared to similar reviews from Cameroon and other countries in the future.

**Knowledge and awareness among the public:** Lack of knowledge and awareness of disease and disability among the public was the most common theme identified in this literature, with several studies addressing knowledge and awareness of epilepsy, and a few others on cancer, diabetes and hypertension. This lack of knowledge affects care-seeking behavior and results in further impairments and disability, disease exacerbations, disease transmission, and social exclusion.

**Stigma:** Stigma towards PLWD was found in 21 studies across multiple disease conditions, including mental health conditions, epilepsy, hearing loss, and mobility impairments. These findings indicate that stigma towards PLWD results in a cycle of discrimination and marginalization. Stigma can be conceptualized as a way to mark groups who are viewed to be 'unworthy' of social investment [62]. PLWD were viewed to be less capable and were excluded from work and social participation in the community. Therefore, they were perceived as contributing less to society and had fewer financial resources, perpetuating stigma against them. A common American theory of stigma holds that stigma is a social process; created as a way to reduce the perceived personal risk regarding loss of one's social identity [63] and economic status [64]. As the risk of status loss for PLWD in Cameroon is high, it is not surprising that people are afraid of such loss and take measures to separate themselves from those who experience it. Thus, stigmatizing beliefs may arise from and contribute to an 'us versus them' mentality [65], with status loss being projected away from the stigmatizer onto the group of people who experience the disability. PLWD are negatively labeled and associated with undesirable characteristics [66]. Stigmatization draws on existing forms of social prejudice and power [64, 65], which in Cameroon appear to include beliefs about the causes of disability. Disability can be perceived as preventable, controllable, or punishment for social/cultural offences or immoral behaviours [39, 59], which are then associated with 'carriers' of the disability [64]. Beliefs that attribute the cause of disability to individual decisions or choices rather than to external factors may provide others with justification for holding stigma and discrimination against the 'wrong doers' of society. A social model of disability [11, 64], provides a way to understand that functioning and participation of PLWD in Cameroon is limited by stigma and discrimination as much as, and perhaps even more so, than the physical and mental limitations posed by the impairment itself. Further studies on disability, stigma, discrimination, and strategies to promote social inclusion are needed.

**Impacts on quality of care:** Research related to mental health, epilepsy, mobility impairments, and HIV suggests that lack of knowledge and stigma was found among healthcare providers including physicians, nurses and traditional healers [20, 24, 26, 31, 38, 55]. Lack of knowledge among healthcare providers can lead to poor quality services, misdiagnosis, inappropriate treatment and delays in treatment leading to poor prognosis for conditions such as cancer [49]. The literature on epilepsy suggests that the level of stigmatizing attitudes held by healthcare providers is comparable to those among the general public; with traditional healers having the highest rates of negative attitudes among healthcare providers [30].

**Social support, intimate relationships and marriage:** The difficulties encountered related to intimate relationships and marriage were addressed in number of studies [24–32, 38, 67], and need to be further explored. While not well explored in these studies, it appears that beliefs about what is best for a family may be prioritized over the needs of a disabled family member, in keeping with the overall collectivist nature of society in Cameroon. The lack of a health insurance and income supports for PLWD also appears to play a role regarding social support, intimate relationships and marriage. For example, the costs involved in biomedical treatment for Buruli Ulcer, including travel to hospital, the caregiver's lost wages, and costs of supporting the admitted patient financially, were much greater when families were involved in patient care [58], which led many families to avoid these costs by socially isolating the patient in the hospital, negatively impacting the patient's well-being [58]. Similarly, mental illness may also lead to a loss of familial relationships. Menick et al. discussed how families abandoned their loved one with mental illness on a main street in Yaoundé, hoping that a 'hypnotist' could heal them [16].

### Gaps in the healthcare systems

*Lack of health workers:* there is a shortage of mental healthcare workers in Sub-Saharan Africa [20, 68]. For example, in 2011 there were 0.03% psychiatrists per 100,000 people in Cameroon, approximately 6 or 7 in the country [69]. Mental healthcare training is lacking: the majority of primary care doctors and nurses have not received in-service training in mental health care within the past five years [69]. Healthcare professionals need education regarding the prevalence, causes, and management of mental health conditions, which could help to reduce stigma, and more research is required to better understand conditions and effective treatments. We did not find any other papers addressing human resources for other disabilities.

*Work, employment, and financial barriers to medical care:* restrictions to employment, and financial barriers to medical consultation and treatment were two key themes identified in this literature. PLWD were refused employment or faced unequal employment opportunities due to social stigma, participation restrictions, and a lack of education [22, 34, 35, 38, 39]. This exclusion from fair work and employment combined with the lack of social assistance for PLWD in Cameroon resulted in poverty and financial barriers. As a result, seeking treatment from medical providers and compliance of medication became second priority.

*Gaps in research:* the perception that there is a lack of research on disability in Africa was supported by the results of our review. Two major gaps were the lack of primary studies focused on disability and the lack of literature reviews of disability research. Of the studies included in this review, few focused on disability, functioning, and participation as the main topic of research investigation. Rather, findings related to functioning and social participation were more often mentioned indirectly, as smaller findings in relation to the main topic of the study. Other gaps of note were identified in the mental health literature. The studies were focused on the prevalence and correlates of mental illness. Only one study directly examined the efficacy of an intervention for mental illness.

## Conclusion

Using the ICF framework and additional themes, information from primary research studies related to disability in Cameroon was collated. Existing research topics and gaps in the literature were identified. This review found that there is a very small body of primary research on the impacts of disability in Cameroon, with no secondary literature reviews identified. A strong need for further disability research exists, especially research that addresses other aspects of disability besides prevalence and correlates alone. We recommended that future research focus on the impacts of disability on functioning and participation in these and other areas of daily life: community participation and contribution; functioning in domestic activities; familial, intimate and other social relationships and dynamics, and the impact of environmental factors. Research should also be conducted that examines the prevalence and impacts of various types of stigma including discrimination, prejudice, and inequality; generally and specific to conditions. Mental health research is needed to examine stigma as well as traditional conceptualizations of mental health and traditional, psychosocial, and biomedical forms of interventions. Future research on the factors is essential to gain greater insight into the challenges experienced by people with disabilities living in Cameroon, as well as to inform decision making in a number of areas, including: disability policy, funding allocation to improve the lives of people with disabilities, clinical services and evidence-based healthcare for people with disabilities, and directions for future research efforts.

### What is known about this topic

- Disability is more prevalent in low-income countries;
- There is limited research on disability and the impact of disability in low-income countries.

### What this study adds

- Disability in Cameroon is linked stigma and discrimination;
- There is a general lack of knowledge and awareness on disability among the public and health workers in Cameroon;
- Disability has an important impact on quality of care and employment.

## Competing interests

The author declare no competing interest.

## References

[1] WHO Disability and health: fact sheet N°352..

[2] WHO Global burden of disease: 2004 update..

[3] WHO Global atlas of medical devices..

[4] UNHCR 2015 UNHCR country operations profile Cameroon..

[5] IMF Cameroon: 2014 Article IV Consultation..

[6] Lantum DN, Mbah D, Fogang JT, Minkoulou MEM Situation Analysis of Health Research in Cameroon..

[7] UNDP Human development reports: Cameroon..

[8] WHO/GHWA Cameroon..

[9] WHO Global health observatory (GHO) data..

[10] Mitra S, Posarac A, Vick BC Disability and poverty in developing countries: a snapshot from the World Health Survey..

[11] WHO International Classification of Functioning..

[12] Ministère de la Santé Publique Cameroun: strategie sectorielle de Sante 2001-2015..

[13] Moher D, Liberati A, Tetzlaff J, Altman DG, Group P (2009). Preferred reporting items for systematic reviews and meta-analyses: the PRISMA statement. PLoS Med..

[14] Gaynes BN, Pence BW, Atashili J, O'Donnell J, Kats D, Ndumbe PM (2012). Prevalence and predictors of major depression in HIV-infected patients on antiretroviral therapy in Bamenda, a semi-urban center in Cameroon. PLoS One..

[15] L'Akoa R M, Noubiap JJ, Fang Y, Ntone FE, Kuaban C (2013). Prevalence and correlates of depressive symptoms in HIV-positive patients: a cross-sectional study among newly diagnosed patients in Yaounde, Cameroon. BMC Psychiatry..

[16] Mbassa Menick D, Menguene Mviena J (2006). Modalités expressives des dépressions en psychopathologie camerounaise. Synapse..

[17] Pence BW, Gaynes BN, Atashili J, O'Donnell JK, Kats D, Whetten K (2014). Feasibility, safety, acceptability, and preliminary efficacy of measurement-based care depression treatment for HIV patients in Bamenda, Cameroon. AIDS Behav..

[18] Mbassa Menick D (2006). Réhabilitation psychosociale des malades mentaux errants: Évaluation d'un dispositif mis en place dans la ville de Yaoundé. Synapse..

[19] Mbassa Menick D (2009). Auteurs d'homicides et de tentatives d'homicides, quelles caractéristiques, etude portant sur des rapports d'expertises psychiatriques faits au Cameroun. Med Trop.

[20] Keugoung B, Kongnyu ET, Meli J, Criel B (2013). Profile of suicide in rural Cameroon: are health systems doing enough. Trop Med Int Health..

[21] St Louis KO, Roberts PM (2013). Public attitudes toward mental illness in Africa and North America. Afr J Psychiatry (Johannesbg)..

[22] Allotey P, Reidpath D (2007). Epilepsy, culture, identity and well-being: a study of the social, cultural and environmental context of epilepsy in Cameroon. J Health Psychol..

[23] Bain LE, Awah PK, Takougang I, Sigal Y, Ajime TT (2013). Public awareness, knowledge and practice relating to epilepsy amongst adult residents in rural Cameroon--case study of the Fundong health district. Pan Afr Med J..

[24] Njamnshi AK, Angwafor SA, Baumann F, Angwafo FF, Jallon P, Muna WF (2009). Knowledge, attitudes, and practice of Cameroonian medical students and graduating physicians with respect to epilepsy. Epilepsia..

[25] Njamnshi AK, Angwafor SA, Jallon P, Muna WF (2009). Secondary school students' knowledge, attitudes, and practice toward epilepsy in the Batibo Health District--Cameroon. Epilepsia..

[26] Njamnshi AK, Angwafor SA, Tabah EN, Jallon P, Muna WF (2009). General public knowledge, attitudes, and practices with respect to epilepsy in the Batibo Health District, Cameroon. Epilepsy Behav..

[27] Njamnshi AK, Tabah EN, Yepnjio FN, Angwafor SA, Dema F, Fonsah JY (2009). General public awareness, perceptions, and attitudes with respect to epilepsy in the Akwaya Health District, South-West Region, Cameroon. Epilepsy Behav..

[28] Njamnshi AK, Yepnjio FN, Bissek AC, Tabah EN, Ongolo-Zogo P, Dema F (2009). A survey of public knowledge, attitudes, and practices with respect to epilepsy in Badissa village, centre region of Cameroon. Epilepsy Behav..

[29] Njamnshi AK, Yepnjio FN, Tabah EN, Dema F, Angwafor SA, Fonsah JY (2009). Public awareness, perceptions, and attitudes with respect to epilepsy in Ebolowa and Sangmelima--Urban Cameroon. Epilepsy Behav..

[30] Njamnshi AK, Bissek AC, Yepnjio FN, Tabah EN, Angwafor SA, Kuate CT (2010). A community survey of knowledge, perceptions, and practice with respect to epilepsy among traditional healers in the Batibo Health District, Cameroon. Epilepsy Behav..

[31] Njamnshi AK, Tabah EN, Bissek AC, Yepnjio FN, Angwafor SA, Dema F (2010). Knowledge, attitudes and practices with respect to epilepsy among student nurses and laboratory assistants in the South West Region of Cameroon. Epilepsy Behav..

[32] Njamnshi AK, Tabah EN, Bissek AC, Yepnjio FN, Kuate C, Angwafor SA (2010). Knowledge, attitudes and practice with respect to epilepsy among secondary school students in the Kumbo West Health District - North West Region- Cameroon. Epilepsy Behav..

[33] Cubo E, Doumbe J, Martinez-Martin P, Rodriguez-Blazquez C, Kuate C, Mariscal N (2014). Comparison of the clinical profile of Parkinson's disease between Spanish and Cameroonian Cohorts. J Neurol Sci..

[34] De Clerck GA (2011). Fostering Deaf People's Empowerment: the Cameroonian deaf community and epistemological equity. Third World Quarterly..

[35] Touko A, Mboua CP, Tohmuntain PM, Perrot AB (2010). Sexual vulnerability and HIV seroprevalence among the deaf and hearing impaired in Cameroon. J Int AIDS Soc..

[36] Oye JE, Kuper H, Dineen B, Befidi-Mengue R, Foster A (2006). Prevalence and causes of blindness and visual impairment in Muyuka: a rural health district in South West Province, Cameroon. Br J Ophthalmol..

[37] Oye JE, Kuper H (2007). Prevalence and causes of blindness and visual impairment in Limbe urban area, South West Province, Cameroon. Br J Ophthalmol..

[38] Bremer K, Cockburn L, Ruth A (2010). Reproductive health experiences among women with physical disabilities in the Northwest Region of Cameroon. Int J Gynaecol Obstet..

[39] Abanda F, Biligha P (2014). Citizenship and vulnerability: The case of the disabled citizen and urbanism policies of the real estate public space in Cameroon. Studia Universitatis Babes-Bolyai Studia Europaea..

[40] Kiani S (2009). Women with disabilities in the North-West province of Cameroon: resilient and deserving of greater attention. Disability & Society..

[41] Nanfosso RAT, Zamo-Akono CM (2010). Fertility, Health and Female Labour Force Participation in Urban Cameroon. International Business Research..

[42] Zamo-akono C (2013). Disability and Labour Force Participation in Cameroon. International Journal of Human Resource Studies..

[43] Kiawi E, Edwards R, Shu J, Unwin N, Kamadjeu R, Mbanya JC (2006). Knowledge, attitudes, and behavior relating to diabetes and its main risk factors among urban residents in Cameroon: a qualitative survey. Ethn Dis..

[44] Awah PK, Kengne AP, Fezeu LL, Mbanya JC (2008). Perceived risk factors of cardiovascular diseases and diabetes in Cameroon. Health Educ Res..

[45] Kamadjeu RM, Edwards R, Atanga JS, Unwin N, Kiawi EC, Mbanya JC (2006). Prevalence, awareness and management of hypertension in Cameroon: findings of the 2003 Cameroon Burden of Diabetes Baseline Survey. J Hum Hypertens..

[46] Fezeu L, Fointama E, Ngufor G, Mbeh G, Mbanya JC (2010). Diabetes awareness in general population in Cameroon. Diabetes Res Clin Pract..

[47] Awah PK, Unwin NC, Phillimore PR (2009). Diabetes Mellitus: Indigenous naming, indigenous diagnosis and self-management in an African setting: the example from Cameroon. BMC Endocr Disord..

[48] Awah PK, Phillimore P (2008). Diabetes, Medicine and Modernity in Cameroon. Africa: Journal of the International African Institute..

[49] Ekortarl A, Ndom P, Sacks A (2007). A study of patients who appear with far advanced cancer at Yaounde General Hospital, Cameroon, Africa. Psychooncology..

[50] Tebeu PM, Major AL, Rapiti E, Petignat P, Bouchardy C, Sando Z (2008). The attitude and knowledge of cervical cancer by Cameroonian women; a clinical survey conducted in Maroua, the capital of Far North Province of Cameroon. Int J Gynecol Cancer..

[51] Boyer S, Clerc I, Bonono CR, Marcellin F, Bile PC, Ventelou B (2011). Non-adherence to antiretroviral treatment and unplanned treatment interruption among people living with HIV/AIDS in Cameroon: Individual and healthcare supply-related factors. Soc Sci Med..

[52] Boyer S, Protopopescu C, Marcellin F, Carrieri MP, Koulla-Shiro S, Moatti JP (2012). Performance of HIV care decentralization from the patient's perspective: health-related quality of life and perceived quality of services in Cameroon. Health Policy Plan..

[53] Jacobi CA, Atanga PN, Bin LK, Mbome VN, Akam W, Bogner JR (2013). HIV/AIDS-related stigma felt by people living with HIV from Buea, Cameroon. AIDS Care..

[54] Marcellin F, Bonono CR, Blanche J, Carrieri MP, Spire B, Koulla-Shiro S (2010). Higher risk of unsafe sex and impaired quality of life among patients not receiving antiretroviral therapy in Cameroon: results from the EVAL survey (ANRS 12-116). AIDS..

[55] Rosenburg N, Taliaferro D, Ercole P (2012). HIV-related stigma among nursing students in Cameroon. J Assoc Nurses AIDS Care..

[56] Suzan-Monti M, Blanche J, Bile P, Koulla-Shiro S, Abu-Zaineh M, Marcellin F (2011). Individual and structural factors associated with HIV status disclosure to main partner in Cameroon: ANRS 12-116 EVAL survey, 2006-2007. J Acquir Immune Defic Syndr..

[57] Yakam AN, Noeske J, Angumua C, Bowong S, Fono LA (2013). Management of tuberculosis patients in the urban setting: Health service delivery and health care-seeking behaviour. Sante Publique (Bucur)..

[58] Grietens KP, Boock AU, Peeters H, Hausmann-Muela S, Toomer E, Ribera JM (2008). "It is me who endures but my family that suffers": social isolation as a consequence of the household cost burden of Buruli ulcer free of charge hospital treatment. PLoS Negl Trop Dis..

[59] Peeters Grietens K, Toomer E, Um Boock A, Hausmann-Muela S, Peeters H, Kanobana K (2012). What role do traditional beliefs play in treatment seeking and delay for Buruli ulcer disease,insights from a mixed methods study in Cameroon. PLoS One..

[60] Amuyunzu-Nyamongo M, Tchounkeu YF, Oyugi RA, Kabali AT, Okeibunor JC, Manianga C (2011). Drawing and interpreting data: Children's impressions of onchocerciasis and community-directed treatment with ivermectin (CDTI) in four onchocerciasis endemic countries in Africa. Int J Qual Stud Health Well-being..

[61] Njelesani J, Couto S, Cameron D (2011). Disability and rehabilitation in Tanzania: a review of the literature. Disabil Rehabil..

[62] Reidpath DD, Chan KY, Gifford SM, Allotey P (2005). "He hath the French pox": stigma, social value and social exclusion. Sociol Health Illn..

[63] Major B, Eccleston CP (2005). Stigma and social exclusion..

[64] Deacon H (2006). Towards a sustainable theory of health-related stigma: lessons from the HIV/AIDS literature. J Community Appl Soc Psychol..

[65] Link BG, Phelan JC (2006). Stigma and its public health implications. Lancet..

[66] Link BG, Phelan JC (2001). Conceptualizing stigma. Annual review of Sociology..

[67] Njamnshi AK, Angwafor SA, Tabah EN, Yepnjio FN, Lekoubou AZ, Dema F (2009). Knowledge, perceptions and practice with respect to epilepsy among traditional healers in the Batibo health district, Cameroon. J Neurol Sci..

[68] WHO Mental Health Atlas 2014..

[69] WHO Mental health atlas 2011: Cameroon..

